# Neurite orientation dispersion and density imaging in stroke: current applications, limitations, and future directions

**DOI:** 10.3389/fnhum.2026.1924031

**Published:** 2026-09-15

**Authors:** Wanxia Wang, Xin Wen, Yuan Zhang, Hongyu Wang, Longhua Hu, Cheng Tan, Zicai Liu

**Affiliations:** 1 Department of Rehabilitation Medicine, Shaoguan University Affiliated Hospital (Shaoguan First People’s Hospital), Shaoguan, China; 2 School of Rehabilitation Medicine, Gannan Medical University, Ganzhou, China; 3 Department of Rehabilitation Therapy Teaching and Research, Gannan Healthcare Vocational College, Ganzhou, China

**Keywords:** diffusion tensor imaging, neurite orientation dispersion and density imaging, neuropsychiatric disorders, post-stroke depression, stroke

## Abstract

Stroke is currently one of the leading causes of death and disability worldwide among neurological diseases. Although traditional imaging techniques can clearly delineate lesion extent and hemorrhagic patterns, they are limited in quantifying microscopic structural changes and repair processes in early-stage brain tissue damage. Neurite Orientation Dispersion and Density Imaging (NODDI), an advanced diffusion-weighted MRI technique, enables accurate, non-invasive quantification of post-stroke microstructural changes in brain tissue. NODDI objectively quantifies neurite damage and remodeling, providing crucial imaging evidence for stroke severity, prognosis, and rehabilitation outcome evaluation. This review critically synthesizes NODDI’s biophysical principles, evaluates its expanding applications in both ischemic and hemorrhagic stroke—highlighting recent advancements in modeling and clinical translation—discusses persistent limitations, and proposes a roadmap for future research to maximize its clinical utility and address current challenges. Importantly, this review focuses exclusively on magnetic resonance imaging (MRI)-based techniques and does not cover non-MRI modalities such as computed tomography (CT) or positron emission tomography (PET). Notably, post-stroke depression (PSD) affects approximately one-third of stroke survivors and represents a major neuropsychiatric complication that impedes functional recovery and reduces quality of life. Emerging evidence demonstrates that NODDI-derived metrics—particularly ODI and ISO—can identify microstructural disruptions in emotion-related white matter pathways that are specific to PSD, offering potential neuroimaging biomarkers that surpass conventional DTI parameters. These findings position NODDI as a promising tool not only for stroke characterization but also for bridging the gap between focal brain injury and subsequent psychiatric manifestations.

## 1 Introduction

Stroke is a highly prevalent acute cerebrovascular disease worldwide, characterized by extremely high incidence, disability, recurrence, and mortality rates (Benjamin et al., 2019). Its core pathological mechanism lies in localized cerebral ischemia and hypoxia caused by acute occlusion or rupture of cerebral blood vessels (Lee et al., 2022). Ischemic stroke accounts for the vast majority of strokes (Pantoni, 2010), and its pathological progression involves a series of complex changes, including cerebral ischemia and hypoxia, neuronal damage, axonal rupture, and white matter microstructural remodeling (Gao et al., 2024; Lo, 2008). Hemorrhagic stroke is similarly associated with significant destruction of nerve fibers and microstructural damage (Zhang et al., 2025). Therefore, the precise and non-invasive assessment of microstructural changes in brain tissue following a stroke holds critical clinical value for elucidating injury mechanisms, guiding personalized treatment, predicting neurological recovery, and evaluating prognosis (Cramer et al., 2011).

Traditional magnetic resonance diffusion imaging techniques, such as DTI, have been widely used to assess white matter damage in stroke (Le Bihan et al., 2001). However, their single-compartment model assumption makes it difficult to distinguish among different microenvironments, such as intracellular, extracellular, and cerebrospinal fluid. This technique is easily compromised in areas with crossing or scattered nerve fibers, and its parameters are susceptible to interference from pathological changes such as edema and inflammation (Jellison et al., 2004), making it difficult to accurately reflect the true density, arrangement, and integrity of axons and dendrites (Jellison et al., 2004; Khalil et al., 2025; Sotak, 2002). In recent years, Neurite Orientation Dispersion and Density Imaging (NODDI), an advanced MRI technique based on diffusion MRI, has distinguished between intracellular and extracellular water diffusion signals through mathematical modeling. It can non-invasively quantify core parameters including the Neurite Density Index (NDI), Orientation Dispersion Index (ODI), free water fraction (FWF), also known as isotropic volume fraction (ISO), and Intracellular Volume Fraction (ICVF) (Zhang et al., 2012). NODDI offers a more specific characterization of axonal and dendritic microstructural features (Seyedmirzaei et al., 2023) and detects early-stage stroke-related microstructural changes often missed by conventional imaging, thus demonstrating superior sensitivity and specificity compared to traditional DTI (Mouchtouris et al., 2024; Wang et al., 2019).

With the gradual adoption of NODDI technology in the study of central nervous system diseases in recent years, its value in the field of stroke has become increasingly evident: Firstly, it provides clinicians with detailed microscopic images of brain tissue in cases of hemorrhagic or ischemic stroke, enabling precise localization of lesion extent and hemorrhagic patterns (Yuan et al., 2025). Furthermore, dynamic monitoring of NDI and ODI indices offers objective evaluation of the efficacy of thrombolysis, thrombectomy, or rehabilitation therapy, as well as the potential for recovery of patients’ motor and cognitive functions (Mouchtouris et al., 2024; Li et al., 2024). This review will provide a systematic overview of the technical principles and parametric significance of NODDI, as well as recent research progress in damage assessment, neurological function prediction, and rehabilitation efficacy evaluation for both ischemic and hemorrhagic strokes. It will analyze the current shortcomings and challenges in this field and discuss the future directions for precision diagnosis and treatment of stroke.

## 2 Technical foundations of NODDI

NODDI proposes a three-compartment biophysical model [12]that, unlike conventional DTI and diffusion kurtosis imaging (DKI), distinguishes between intracellular, extracellular, and cerebrospinal fluid spaces (Yu et al., 2024; Parker et al., 2023) (as shown in Figures 1–3). Intracellular Compartment (IC): Represents the space enclosed by axonal membranes and possibly dendritic membranes, modeled as a set of infinitely thin rods with zero radius. It assumes that the height of water molecule diffusion perpendicular to the direction of the neurites is highly restricted, while diffusion along the direction of the neurites is unimpeded. Its directional distribution ranges from highly parallel to highly dispersed, and the Watson distribution (an axisymmetric distribution characterized by a single parameter) is used to describe the degree of dispersion along the neurite direction (Kamiya et al., 2020; Schneider et al., 2017; Grussu et al., 2017). Extracellular Compartment (EC): This encompasses all tissues other than the axon and cerebrospinal fluid (CSF). Water molecule diffusion is hindered by the surrounding tissue structure but not completely restricted, and is described using an anisotropic Gaussian diffusion model (Kamiya et al., 2020; Schneider et al., 2017). Cerebrospinal fluid compartment (CSF): Represented by an isotropic Gaussian diffusion model, simulating the space occupied by cerebrospinal fluid, where water molecule diffusion is not restricted by direction (Schneider et al., 2017; Grussu et al., 2017).

**Figure 1:**
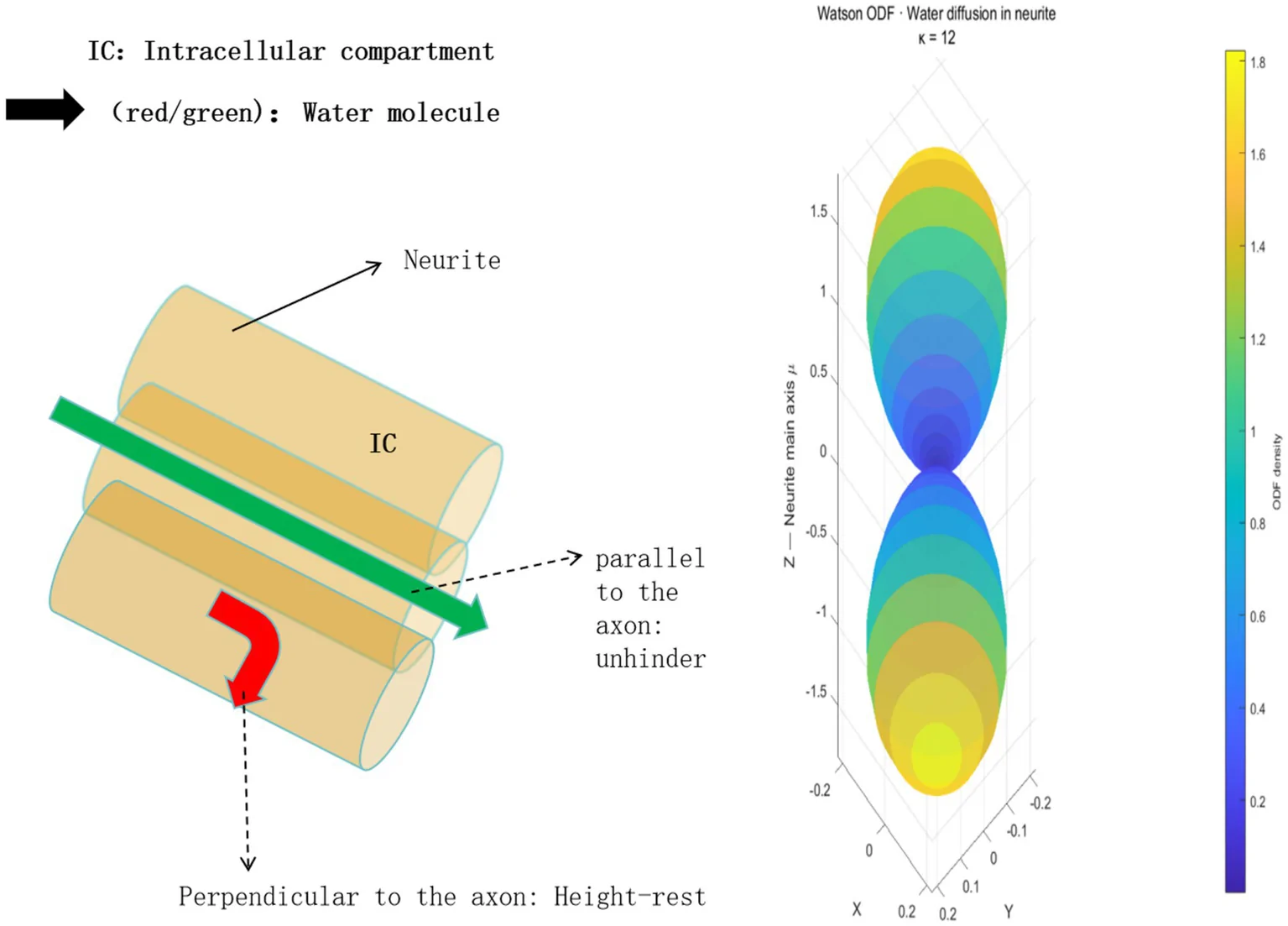
Intracellular compartment (IC). Modeled as zero-radius cylinders representing neurites, with restricted diffusion perpendicular to and free diffusion along the neurite orientation. Directionality is described by a Watson distribution.

**Figure 2:**
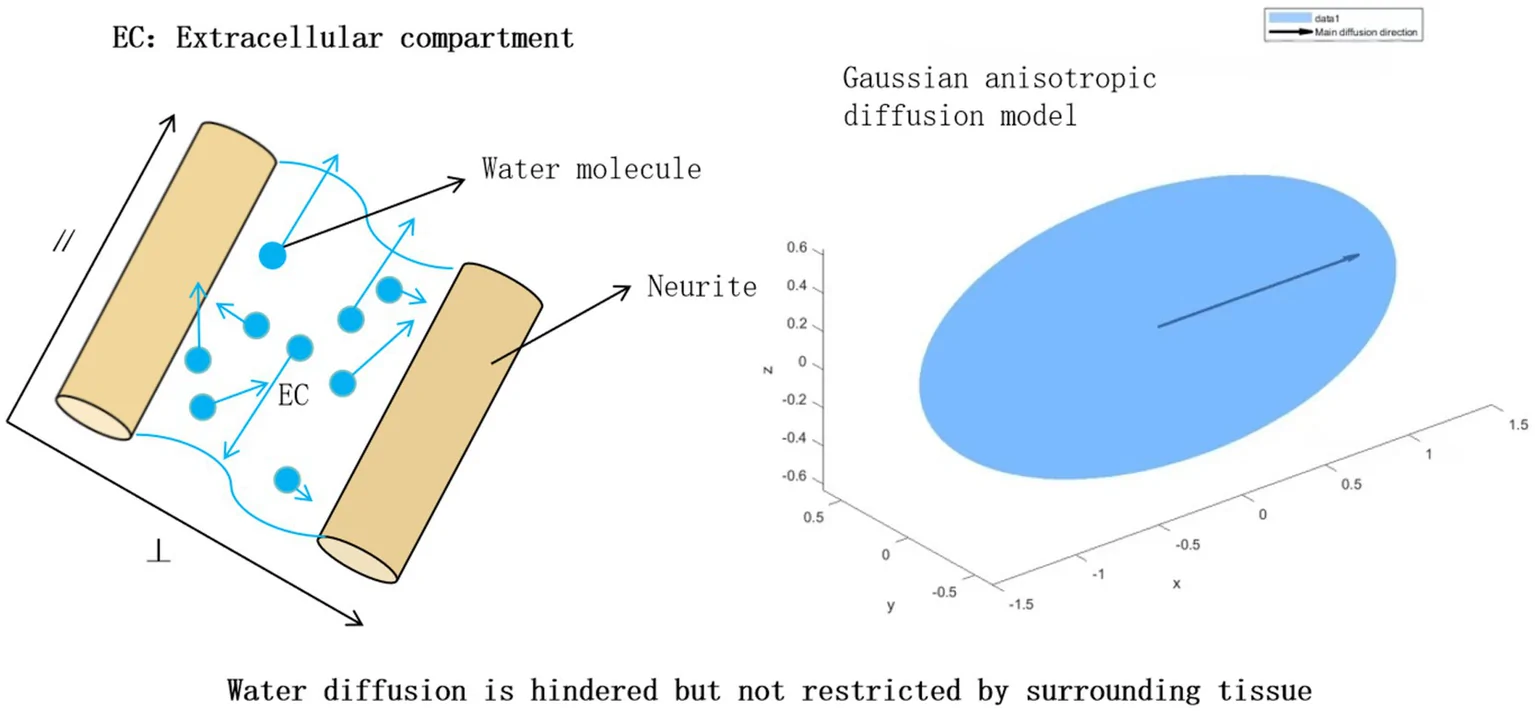
Extracellular compartment (EC). Water diffusion is hindered but not restricted by surrounding tissue; a simple (Gaussian) anisotropic diffusion model is used.

**Figure 3:**
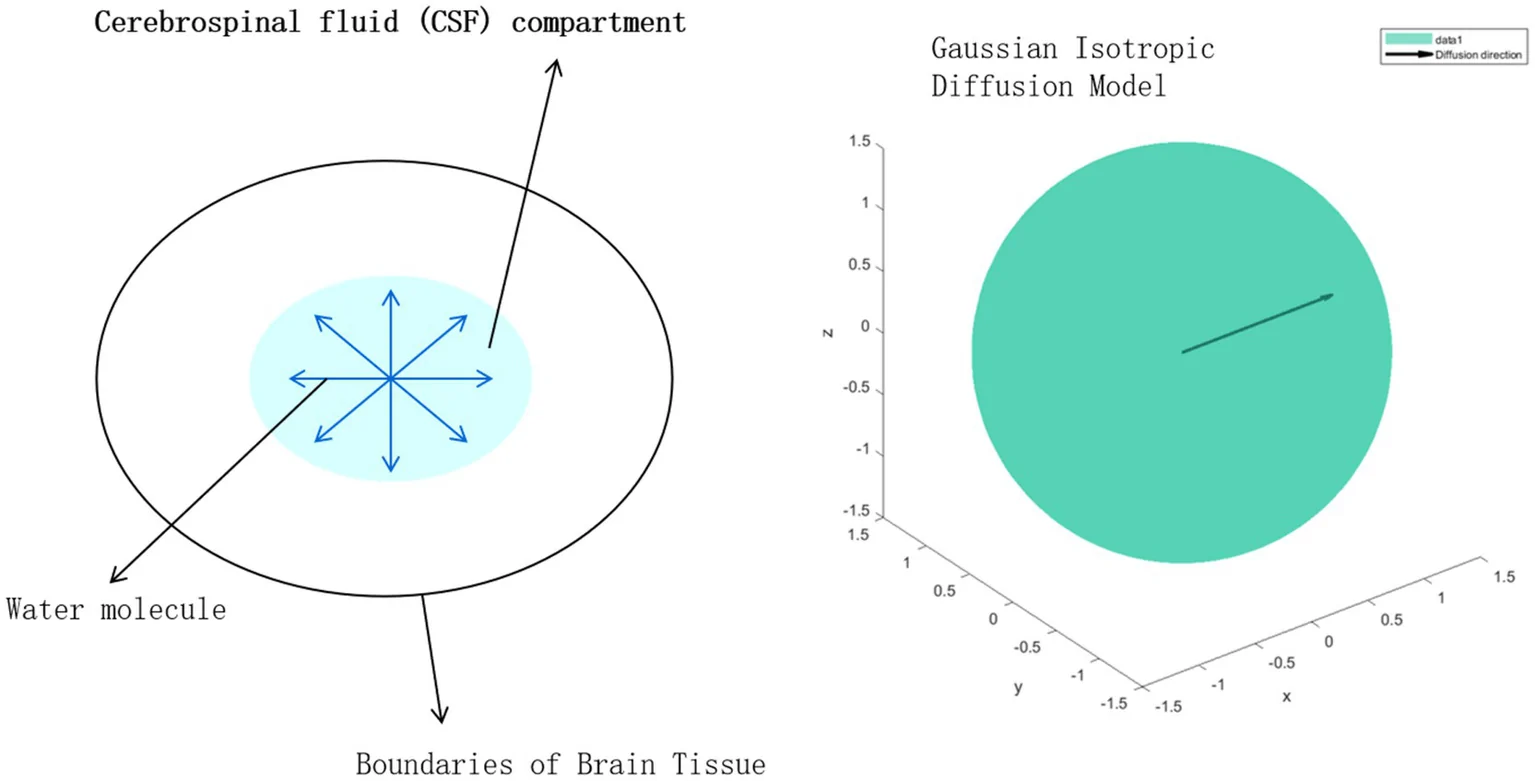
Cerebrospinal fluid (CSF) compartment. The volume occupied by CSF is modeled using an isotropic Gaussian diffusion model.

NODDI provides unique information regarding neurite density, directional dispersion, and the proportion of unrestricted (“free”) water within voxels (Parker et al., 2023; Karamched et al., 2025). Among these, the NODDI-derived neurite density index (NDI), frequently interpreted as the intracellular volume fraction (ICVF), provides an estimate of neurite density, a higher NDI/ICVF value indicates greater neurite density (Schifani et al., 2025). In stroke patients, a lower NDI value correlates with more severe neurite damage (Legouhy et al., 2023). Clinically, the extent and severity of brain injury can be assessed by measuring NDI values in different regions. A sustained decrease in NDI values following onset often indicates disease progression, whereas a gradual increase may suggest some degree of neural tissue repair. ODI reflects the degree of dispersion in the orientation of axons; in normal brain tissue, nerve fibers are arranged in an orderly manner, resulting in relatively low ODI values, whereas ODI values increase following a stroke (Legouhy et al., 2023). For stroke patients, monitoring changes in ODI values allows for an understanding of the disruption of nerve fibers and an assessment of disease severity. If ODI values continue to rise and the affected area expands, this suggests worsening neural damage; conversely, if ODI values gradually decrease and return toward normal levels, this may indicate that the structure of nerve fibers is gradually recovering. FWF/ISO reflects the free water fraction/isotropic volume fraction, representing unrestricted free water in the interstitial spaces that diffuses isotropically. Higher values indicate a greater amount of free water. This parameter specifically reflects the degree of brain tissue edema, inflammatory response, and microstructural damage. Elevated values are directly associated with vasogenic edema following ischemic stroke, inflammatory activity caused by blood–brain barrier damage, and microstructural damage to the brain parenchyma resulting from ischemia (Mouchtouris et al., 2024; Diamandi et al., 2025; Palacios et al., 2020) (As shown in Table 1).

**Table 1:** Definitions, physiological significance, and clinical significance of key NODDI parameters.

Parameters	Physiological significance	Clinical significance
NDI	Reflects the number and density of neural processes (axons and dendrites) per unit volume; the higher the value, the denser the neural processes.	Assess the extent of neuronal damage, neuronal loss, and atrophy following a stroke; monitor neuronal regeneration/repair.
ODI	Indicates the degree of disorder or orderliness in the direction of nerve fiber tracts. A higher value indicates more disordered fiber arrangement, more crossings, and a more dispersed direction; a lower value indicates the opposite.	Indicates fiber bundle disruption, demyelination, and structural disorganization; assesses the integrity of white matter microstructure
FWF/ISO	Represents the unbound free water in the interstitial spaces, which diffuses isotropically. The higher the value, the greater the amount of free water.	Indicates edema, inflammation, and tissue damage

NDI, neurite density index; ODI, orientation dispersion index; FWF/ISO, free water fraction/isotropic volume fraction.

## 3 Current applications of NODDI technology in the field of stroke

NODDI has been increasingly applied to stroke research across multiple domains, including white matter integrity assessment, treatment monitoring, and the characterization of post-stroke neuropsychiatric complications. In the following subsections, we synthesize current evidence spanning from preclinical models to clinical cohorts, illustrating both the versatility of NODDI and the methodological considerations required for its valid interpretation.

In terms of white matter, its integrity is crucial for brain function. However, white matter has limited collateral circulation and blood supply and is highly sensitive to ischemia; consequently, stroke often leads to white matter damage, severely impairing neural function and recovery (Guo et al., 2025). In 2014, Adluru et al. (2014) first applied four-shell high-angular resolution diffusion imaging (HARDI) data to investigate NODDI-derived microstructural changes in the white matter of stroke patients. The study preliminarily indicated that conventional imaging techniques may be nonspecific when measuring the integrity of white matter tracts in stroke patients, whereas NODDI modeling can provide specific information regarding microstructural changes in post-stroke tissue. It can serve as a specific surrogate marker for white matter integrity in stroke patients and may offer deeper insights into post-stroke white matter reorganization (Adluru et al., 2014; Hodgson et al., 2019).

Regarding white matter recovery, ICVF, which quantifies the volume fraction of intracellular space, reflects cellular density. Elevated ICVF is often observed in conditions of cellular swelling, a common pathological response post-stroke. Bagdasarian et al. (2021) demonstrated that human bone marrow-derived mesenchymal stem cells (hMSCs) therapy could mitigate ischemic cellular swelling by stabilizing ICVF, and simultaneously preserve axonal structures and promote orderly repair through stable ODI values. This approach also offers a more sensitive basis for assessing or predicting treatment response. For gray matter recovery, although hMSC treatment did not result in significant differences in gray matter DTI metrics compared to the control group, ICVF indicated that it alleviated gray matter cell swelling. Furthermore, ODI showed that the orientation of gray matter axons returned to normal levels 21 days after treatment. In contrast, the control group failed to achieve this, fully demonstrating NODDI’s precise representation of therapeutic benefits in the gray matter.

Mechanical thrombectomy (MT) has been widely adopted for stroke patients. Favorable functional outcomes following thrombectomy are often accompanied by symptomatic hemorrhagic transformation. The blood–brain barrier (BBB) is compromised by initial ischemic injury, leading to increased BBB permeability; This increased permeability allows blood and inflammatory cells to breach the barrier and extravasate, ultimately elevating the patient’s risk of hemorrhagic transformation (Spronk et al., 2021; Arba et al., 2020). In a 2024 study by Mouchtouris et al. (2024), the combined use of NODDI and Dual-pseudocontinuous arterial spin labeling (DP-pCASL) was employed to investigate in depth the effects of MT on various tissue compartments surrounding the BBB complex. The study utilized NODDI to obtain parameters such as ICVF, FWF, and ODI, further elucidating the association between changes in blood–brain barrier function following MT and microstructural damage in the brain parenchyma. This enabled a precise assessment of microstructural changes in different brain tissue compartments, providing multidimensional evidence for understanding the impact of MT on blood–brain barrier integrity.

Beyond motor and cognitive sequelae, stroke frequently disrupts limbic–cortical white matter pathways that regulate emotion, predisposing survivors to neuropsychiatric complications. Regarding post-stroke depression (PSD), studies have shown that approximately 30% of stroke survivors suffer from PSD (Hong et al., 2020). PSD not only slows the rate of neurological recovery but also reduces quality of life and even significantly increases mortality among stroke survivors (Feng et al., 2024). Regarding the structural and functional changes associated with post-stroke depression, a 2025 study by Lu et al. (2025) conducted a comparative analysis of NODDI metrics in populations with PSD, stroke without depression (non-PSD), and healthy controls. Through statistical validation and clinical correlation analysis, they concluded that NODDI can accurately identify micro-abnormalities in emotion-related white matter pathways in PSD patients, comparing ODI and ISO across the three groups. The results showed that, compared with non-PSD patients, PSD patients exhibited significantly elevated ODI in the aforementioned pathways, accompanied by significantly elevated ISO, reflecting impaired neural tissue integrity. In contrast, traditional DTI metrics (such as FA and MD) showed no significant differences between the PSD and non-PSD groups, indicating that NODDI can capture PSD-specific microstructural changes that conventional techniques cannot detect. Thus, the application of NODDI to PSD exemplifies how microstructural imaging can extend beyond characterizing focal injury to address clinically important neuropsychiatric sequelae that are integral to post-stroke functional outcomes.

Although NODDI has been increasingly applied to both ischemic and hemorrhagic stroke, the underlying pathophysiological mechanisms and the corresponding NODDI parameter alterations differ substantially between the two entities. In ischemic stroke, the primary insult is cerebral hypoperfusion, leading to cytotoxic edema, energy failure, and subsequent axonal and dendritic injury (Wang et al., 2019). NODDI parameters typically show a rapid decrease in NDI/ICVF within the ischemic core, reflecting acute neurite loss, alongside an increase in FWF/ISO due to vasogenic edema in the penumbra. ODI changes are more variable, often increasing in regions with fiber disorganization but may remain stable or even decrease in severely compressed areas. These metrics have been successfully employed to monitor the evolution of ischemic damage, assess the efficacy of reperfusion therapies, and track stem cell-mediated neurorepair (Mouchtouris et al., 2024; Bagdasarian et al., 2021). In hemorrhagic stroke, by contrast, the primary pathology is mechanical compression and mass effect from the hematoma, along with secondary toxic injury from blood breakdown products, which disrupts both white matter tracts and cortical gray matter (Wang et al., 2025). NODDI reveals a distinct pattern: NDI is markedly reduced in peri-hematomal regions due to compression-induced neurite loss and Wallerian degeneration, while ODI often increases as fiber tracts are pushed aside and disorganized. Notably, these microstructural alterations may extend to remote areas, such as the thalamus and corticospinal pathways, correlating with cognitive and motor deficits, as demonstrated in thalamic stroke patients (Zhang et al., 2025). Furthermore, FWF/ISO elevation in hemorrhagic stroke reflects not only vasogenic edema but also inflammatory response to blood degradation, which follows a different temporal trajectory compared with ischemic stroke (Farrher et al., 2025). Clinically, NODDI in hemorrhagic stroke is particularly valuable for evaluating the extent of perihematomal damage, predicting recovery potential after surgical evacuation, and differentiating reversible compression from irreversible axonal injury (Wang et al., 2025). In contrast to ischemic stroke, where NODDI is often used to guide acute reperfusion decisions and early prognostic stratification, its role in hemorrhagic stroke currently emphasizes chronic phase assessment and rehabilitation monitoring. Therefore, the interpretation of NODDI parameters must be contextualized according to stroke type, lesion age, and the predominant pathophysiological process, as the same numerical change may carry different prognostic implications in ischemic versus hemorrhagic settings Table 2.

**Table 2:** Applications of NODDI in stroke.

Research	Key findings	Importance
(Adluru et al., 2014), Stroke patients (2 cases)	The NODDI index specifically reflects changes in white matter microstructure and is more sensitive to post-stroke white matter reorganization than FA	First demonstration of NODDI’s utility in stroke, revealing superior sensitivity for white matter microstructural changes compared to FA
(Bagdasarian et al., 2021), (rat ischemic stroke model, 16 rats)	hMSCs protect white matter/gray matter microstructure, reduce cellular swelling (decreased ICVF), and stabilize axon orientation (normalized ODI)	Pioneering use of NODDI for quantifying hMSC therapy effects, establishing ICVF as a potential biomarker for cellular swelling and repair mechanisms
(Mouchtouris et al., 2024), Patients with acute ischemic stroke (21 cases)	Elevated MT and CBF levels are significant predictors of increased k_n_,suggesting more intact BBB function.	Novel multimodal approach combining NODDI and DP-pCASL to elucidate BBB integrity changes post-MT, offering insights into microstructural correlates of perfusion.
(Lu et al., 2025), stroke patients (60 cases)	NODDI outperforms DTI in diagnosing PSD	Identifies NODDI’s superior ability over DTI in detecting specific white matter microstructural abnormalities in PSD, providing potential neuroimaging biomarkers for diagnosis.

In summary, research on the application of NODDI technology in the field of stroke has progressively demonstrated its unique advantages and clinical value compared to traditional imaging techniques. Since Adluru et al. first applied this technology to assess the microstructure of white matter in stroke patients in 2014, subsequent studies by Bagdasarian et al., Mouchtouris et al., and Lu et al. have expanded its scope from simple white matter damage assessment to monitoring the efficacy of stem cell therapy, multimodal analysis of the effects of mechanical thrombectomy on the blood–brain barrier, and the identification of specific microstructural abnormalities in post-stroke depression. All these studies have confirmed that NODDI can accurately quantify microstructural changes in white and gray matter following stroke—including axonal density, directional dispersion, and tissue edema—through core parameters such as ICVF, ODI, and FWF/ISO. However, these studies still have many limitations. Early studies had extremely small sample sizes (e.g., Adluru et al. included only 2 patients), which may lead to biased results and a lack of statistical representativeness. Furthermore, some studies used animal models (e.g., Bagdasarian et al.’s rat model), and cross-species differences still need to be validated in human stroke populations. Subsequent clinical studies have also predominantly employed single-center designs, lacking large-sample, and multicenter validation. Furthermore, most studies were cross-sectional observations or short-term follow-ups (e.g., Bagdasarian et al. followed patients for only 21 days); long-term follow-ups were not conducted, nor were stratified analyses performed based on stroke subtypes or clinical scores. Consequently, it is difficult to establish a clear association between these parameters and long-term neurological outcomes, severely limiting the clinical application and translation of this technology.

## 4 Comparison of NODDI with other traditional imaging techniques

This review strictly focuses on MRI-based techniques, so the following comparisons only involve MRI modalities, excluding CT or PET. In diffusion MRI methods, DTI has been widely used to assess post-stroke white matter integrity through parameters like fractional anisotropy (FA) and mean diffusivity (MD). However, DTI relies on a single-compartment Gaussian model, which cannot differentiate signals from intracellular, extracellular, and free water, and is particularly insensitive in areas with fiber crossings or dispersion, as well as in gray matter (Li et al., 2024; Reveley et al., 2024). Diffusion-weighted imaging (DWI) quantifies ischemic lesions via apparent diffusion coefficient (ADC), offering high sensitivity, but it does not take diffusion anisotropy into account and might miss small or hidden lesions (Winston, 2012; Alhaj Omar et al., 2025). Perfusion-weighted imaging (PWI) reflects tissue microvascular distribution and blood flow perfusion, providing information on brain tissue hemodynamics (Meng et al., 2004), and can also help identify the ischemic penumbra in hyperacute and acute ischemic stroke patients. After thrombolysis or other treatments, PWI can assess treatment effectiveness and patient prognosis by observing how perfusion recovers (Kitagawa et al., 2001; Bardutzky et al., 2005).

More advanced diffusion sequences, like DKI and Mean Apparent Propagator MRI (MAP MRI), are more sensitive to tissue complexity and non-Gaussian diffusion (Özarslan et al., 2013). DKI is better at detecting changes in tissue complexity by measuring the non-Gaussian behavior of water diffusion, though its biological interpretation is not very specific (Huang et al., 2022). MAP MRI can reconstruct the entire diffusion propagator without assuming a specific compartment model, giving a detailed picture of diffusion displacement, but it requires high angular and high b-value sampling and cannot directly separate intracellular from extracellular contributions (Benjamini et al., 2019; Avram et al., 2016).

In comparison, NODDI uses a three-compartment biophysical model that can provide axon and dendrite microstructure metrics with clear biological explanations. It has shown better sensitivity for changes in both white and gray matter after a stroke, and several studies (involving stem cell therapy, mechanical thrombectomy, and post-stroke depression) have confirmed its value (Mouchtouris et al., 2024; Bagdasarian et al., 2021; Lu et al., 2025).

However, compared to other MRI techniques, NODDI also has several notable drawbacks, including prolonged acquisition times, inherent model assumptions (e.g., uniform T2 relaxation across compartments), and sensitivity to scanning parameters such as echo time and field strength. These limitations, along with currently proposed solutions, are discussed in detail in Section 5. Therefore, although NODDI offers unique microstructural specificity, its clinical use must consider these practical constraints, and it is often employed alongside conventional MRI sequences for comprehensive stroke evaluation.

## 5 Limitations and improvements in the application of NODDI technology for stroke

NODDI was originally designed for dMRI data acquired using multi-shell linear tensor encoding, which requires signals at multiple b-values and encoding directions (Chung et al., 2016). In terms of acquisition, it requires multiple b-values and encoding directions, usually taking 10–20 min, much longer than conventional DWI (2–3 min) and DTI, which increases the risk of motion artifacts in stroke patients with impaired consciousness or hemiplegia (Zhang et al., 2012; Diamandi et al., 2025). Additionally, the model assumes uniform T2 relaxation time across compartments, making it unable to distinguish between axons and dendrites or capture fine structures like dendritic spines. It also tends to overestimate cerebrospinal fluid fractions in white matter regions, causing NDI values to deviate from physiologically reasonable ranges (Alsameen et al., 2023). The core metric NDI is highly dependent on echo time (TE), significantly increasing as TE lengthens, while DTI metrics like FA remain relatively stable with TE changes (Gong et al., 2020). NODDI parameters are sensitive to magnetic field strength and scanning equipment, affecting the reliability of cross-site or longitudinal comparisons (Chung et al., 2016; Parvathaneni et al., 2018). Finally, although NODDI can detect abnormalities (like decreased NDI or increased ODI), it cannot directly identify the pathological cause; whether it’s demyelination, axonal loss, or abnormal sprouting, confirmation through histopathology or other imaging techniques is still needed (Palacios et al., 2020; Timmers et al., 2015).

To address these issues, Alsameen et al. (2023) refined NODDI model assumptions by developing Constrained NODDI (C-NODDI), which replaces the free water fraction (Fiso) with pre-segmented input from the FSL-FAST algorithm, simplifying the model to two compartments (intracellular and extracellular) without increasing scan time. They conducted a white matter imaging study on 58 cognitively normal adults, comparing the parameter differences, age-related trends, biomarker correlations, and TE sensitivity between C-NODDI and the original NODDI. This brought NDI values back within a physiologically reasonable range, reducing them by 20–40% compared to the original NODDI, and significantly lowering TE sensitivity. In 2020, Gong et al. (2020) developed Multi-Echo NODDI (MTE-NODDI), which integrates data from different TE scans to effectively separate diffusion differences from T2 relaxation differences, further enhancing parameter stability.

Different anatomical structures and biological environments may require different models, making model selection complex. In the NODDI model, the assumption of multiple Gaussian compartments neglects important factors such as exchange between compartments and non-Gaussian effects within compartments (Colgan et al., 2016). Within the axonal compartment, water diffusion is strictly directional and can only occur along the axis of the axon. In the extra-axonal compartment, water diffusion is less restricted, allowing for both axial and radial diffusion. In the free-fluid compartment, water diffusion is completely unconstrained, enabling free movement in all directions (Kellner et al., 2022). Different MRI field strengths exhibit varying sensitivities to water diffusion signals. This implies that if stroke patients undergo follow-up examinations at different hospitals using different devices, changes in their NODDI parameters may not reflect true pathological changes (e.g., a decrease in NDI could result from device differences rather than further loss of axons) and thus cannot be used for dynamic assessment of disease progression (Chung et al., 2016; Parvathaneni et al., 2018). Furthermore, while NODDI can distinguish between NDI and ODI, it cannot directly determine the pathological cause of the abnormality. For example, low NDI and high ODI in study patients may result from various mechanisms, such as myelin abnormalities, delayed axonal pruning, or axonal sprouting (Palacios et al., 2020); NODDI cannot independently differentiate these and requires further validation through histopathology or other imaging techniques (Timmers et al., 2015).

Therefore, in response to the complexity of model selection arising from different anatomical structures and biological environments, as well as the inherent limitations of the NODDI model in terms of assumptions, device dependency, and pathological specificity, subsequent studies have proposed several targeted improvement strategies: In 2025, Farrher et al. (2025) proposed multi-echo NODDI (MTE-NODDI), which, by releasing the fixed intrinsic diffusion coefficient and correcting T2 relaxation-weighted bias, enables the simultaneous quantification of diffusion and relaxation properties in post-stroke ischemic tissue, thereby eliminating the interference of device variability in dynamic assessments; In 2025, Niu et al. (2025) published a review on infant brain development that systematically summarized the current status and challenges of NODDI in neurodevelopmental research, explicitly pointing out the need to cross-validate the biological significance of NDI and ODI by integrating multimodal techniques (such as myelin imaging and histopathology). Meanwhile, the improved Bingham-NODDI model proposed by Tariq et al. (2016) in 2016 replaced the traditional Watson distribution with the Bingham distribution, more accurately describing the dispersion characteristics of complex fiber orientations and improving the precision of parameter estimates in cross-fiber regions. Collectively, these studies have further improved the efficiency and stability of NODDI parameter estimation from three dimensions—model optimization, computational innovation, and multimodal fusion—thereby enhancing its practicality and reliability in the evaluation of brain microstructure and clinical assessment of stroke.

Even with the methodological improvements mentioned above, we still think there are several basic issues holding back the widespread clinical use of NODDI in stroke. Improved models like C-NODDI cut down on some biases but bring in new problems. C-NODDI totally ignores the free water compartment, which could underestimate cerebrospinal fluid in areas with heavy edema or atrophy, and the new models do not make multi-shell scans any faster (Alsameen et al., 2023). MTE-NODDI needs multi-echo scans, which makes scanning longer and less practical in acute stroke (Gong et al., 2020). Bingham-NODDI can better sort out crossing fibers, but it’s computationally heavy and lacks standardized setups across devices (Tariq et al., 2016). So, a single universal model probably will not work for all stroke types or stages; you should pick the model based on the actual clinical situation. Plus, most validation studies are still retrospective and single-center, with different scan protocols, mostly tested on small groups of healthy people or animal stroke models, with no big enough, stratified stroke population data to back it up, making direct meta-analyses or reliable normal reference values impossible.

To overcome these difficulties, we propose developing an adaptive model selection framework that can automatically choose the most suitable NODDI variant (such as standard, C-NODDI, or MTE-NODDI) using machine learning classifiers trained on multicenter data across different tissue types (white matter and gray matter), lesion age, and degree of edema. Data acquisition could also be accelerated using deep learning-based super-resolution reconstruction or generating NODDI parameters from conventional DWI protocols, reducing motion artifacts and making it more applicable to non-cooperative patients. Additionally, efforts could be made to establish a globally open standardized phantom and *in vivo* database covering different field strengths and scanning platforms (Mushtaha et al., 2021; De Luca et al., 2025), paired with AI-driven harmonization algorithms (like ComBat or GANs) to correct device-related parameter inaccuracies, enabling reliable longitudinal and multicenter comparisons (Pinto et al., 2025; Jodoin et al., 2025). Combining NODDI with quantitative T2 mapping, magnetization transfer imaging, and FLAIR can help differentiate edema, demyelination, and axonal loss, improving pathological specificity without fully relying on histopathology (York et al., 2022). We also encourage future researchers to carry out multicenter prospective longitudinal stroke cohort studies: stratifying patients by stroke type (ischemic/hemorrhagic), disease stage (hyperacute/acute/subacute/chronic), and rehabilitation interventions, with long-term sequential NODDI follow-ups. Based on cohort data, reference threshold ranges for NDI, ODI, and FWF can be established according to specific diseases, and standardized diagnostic cutoffs for stroke severity, rehabilitation prognosis, and post-stroke depression screening can be formulated to promote the clinical application of various improved NODDI techniques.

## 6 Conclusions and future prospects

NODDI technology enables precise quantification of gray and white matter microstructures, addressing the limitations of traditional imaging techniques in capturing microscopic structural changes. It demonstrates unique advantages, particularly in the assessment of white matter damage and the detection of gray matter microstructures. In clinical applications, NODDI can effectively assess microscopic damage to brain tissue in both ischemic and hemorrhagic strokes. Neuropil necrosis caused by ischemia and the decrease in neuropil density resulting from compression by hemorrhagic hematomas can both be intuitively visualized through reduced NDI and increased ODI, providing critical evidence for determining lesion extent, disease progression, and the degree of neurological recovery.

The identification of PSD-specific microstructural abnormalities further underscores the translational relevance of NODDI beyond conventional motor outcomes, reinforcing the view that post-stroke imaging should encompass both sensorimotor and affective circuitry. Moreover, extending NODDI applications to post-stroke neuropsychiatric disorders—particularly PSD, anxiety, and cognitive impairment—represents a promising frontier. By quantifying microstructural disruptions in limbic–cortical circuits, NODDI may eventually inform personalized mental health interventions alongside motor rehabilitation, thereby addressing the holistic burden of stroke sequelae.

In the future, research in this field will mainly focus on improving biophysical models, standardizing scanning protocols, and eliminating device-dependent parameter biases to make NODDI parameters more accurate and stable. Creating a global, multi-center standardized NODDI imaging database that includes different magnetic field strengths and scanner models, along with AI calibration, can help keep measurements consistent across devices, which is really helpful for tracking microstructural damage after a stroke over time. At the same time, refining multi-compartment models like MTE-NODDI and Bingham-NODDI can reduce biases from the original NODDI assumptions and make measuring microstructures in gray matter and areas with complex crossing fibers more accurate. Besides, combining NODDI with perfusion imaging, myelin imaging, and biomarkers to create a multimodal collaborative assessment system is likely to become an important research trend. This kind of integration can make up for the weaknesses of single parameters when it comes to pathological specificity, helping to classify the severity of stroke damage, guide personalized treatment, and predict long-term functional outcomes. As technology advances and large-scale multicenter clinical validations pile up, NODDI is expected to overcome its current inherent limitations and become a key quantitative microstructural imaging biomarker, pushing stroke diagnosis and treatment toward precise, microscopic-level healthcare.

Finally, a critical question is whether NODDI has revealed genuinely novel pathophysiological mechanisms beyond what is already known from histology and conventional imaging. We argue that NODDI’s primary contribution is not the discovery of previously unknown biological processes, but rather the non-invasive, *in vivo* decomposition of composite diffusion metrics into biologically interpretable components. Specifically, whereas DTI-derived fractional anisotropy (FA) conflates axonal density, directional dispersion, and myelin integrity—making its pathological interpretation ambiguous—NODDI disentangles these factors into NDI (density) and ODI (dispersion). This distinction has yielded novel insights: in ischemic stroke, NODDI has shown that the ischemic core exhibits a sharp NDI reduction (reflecting axonal loss), while peri-infarct regions show ODI elevation (reflecting fiber disorganization)—a differential pattern that FA alone cannot resolve. In hemorrhagic stroke, NODDI has identified that peri-hematomal NDI reduction extends to remote thalamocortical pathways, correlating with cognitive deficits that are not predictable from hematoma volume alone. Most importantly, in post-stroke depression, NODDI has detected microstructural abnormalities in emotion-related white matter pathways where DTI metrics (FA/MD) showed no significant differences, demonstrating its unique sensitivity to clinically relevant but conventionally “invisible” pathology. Thus, while NODDI does not overturn established stroke pathophysiology, it provides a clinically translatable tool for quantifying microscopic damage with greater specificity, for monitoring treatment responses (e.g., stem cell therapy), and for identifying imaging biomarkers of neuropsychiatric complications. These capabilities, we contend, justify continued investment in its development, standardization, and multicenter validation.

## Glossary

BBB: Blood–brain barrier
CBF: Cerebral blood flow
CSF: Cerebrospinal fluid
DKI: Diffusion kurtosis imaging
DTI: Diffusion tensor imaging
DWI: Diffusion-weighted imaging
FA: Fractional anisotropy
FWF: Free water fraction
HARDI: High-angular resolution diffusion imaging
hMSCs: Human bone marrow-derived mesenchymal stem cells
ICVF: Intracellular volume fraction
ISO: Isotropic volume fraction
MD: Mean diffusivity
MT: Mechanical thrombectomy
NDI: Neurite density index
NODDI: Neurite orientation dispersion and density imaging
ODI: Orientation dispersion index
PSD: Post-stroke depression
TE: echo time
MAP MRI: mean apparent propagator MRI
